# The reproducibility of structured functional assessments in a social security setting: a pre-specified explanatory analysis of the RELY-studies

**DOI:** 10.3389/fpsyt.2025.1643221

**Published:** 2026-01-02

**Authors:** Regina Kunz, Stephanie Giezendanner, David Y. von Allmen, Joerg Jeger, Martin Eichhorn, Ulrike Hoffmann-Richter, Katrin Fischer, Wout de Boer

**Affiliations:** 1 Evidence-based Insurance Medicine (EbIM), Research & Education, Division of Clinical Epidemiology, Department of Clinical Research, University of Basel, Basel, Switzerland; 2 MEDAS Central Switzerland, Luzern, Switzerland; 3 Mental Health Practice, Basel, Switzerland; 4 Swiss National Accident Insurance Funds, Lucerne, Switzerland; 5 Institute Humans in Complex Systems, School of Applied Psychology, University of Applied Sciences Northwestern Switzerland, Olten, Switzerland

**Keywords:** disability insurance, work capacity evaluation, reproducibility of results, evidence-based medicine, mental disorders, international classification of functioning, disability and health, independent medical evaluation, Mini-ICF-APP

## Abstract

**Introduction:**

Limitations in work capacity (WC) need to be quantified in a transparent and reproducible way when insurers of social security decide whether an individual is entitled to disability benefits and to what extent. Structured assessments of work-related physical, mental and social functioning might provide an empirical basis for judgments on residual work capacity (rWC) which determines entitlement to disability benefits. This study examined the functional assessments themselves, their reliability and expert agreement when applied to claimants with mental disorders, and analyzed their relationship to rWC judgments.

**Material and methods:**

We used RELY-data on the reproducibility of rWC judgments. A pool of 40 psychiatric experts interviewed 55 claimants for disability benefits. Interviews were videotaped and watched by three observing psychiatric experts, resulting in 280 individual ratings. All independently rated claimants’ impairments in work-related mental functions and capacity limitations using the Instrument for Functional Assessment in Psychiatry (IFAP-1 mental functions, IFAP-2a/-2b functional capacities related to the last job and alternative work, scaled 0=none to 4=worst) based on the Mini-ICF-APP, and judged rWC (in Switzerland, scaled 100% to 0%) for the last job and suitable alternative work. Analysis for reliability (ICC, intraclass-correlation coefficient) included a two-way random-effects and a linear mixed-effects model. Expert agreement was estimated as standard error of measurement, SEM.

**Results:**

The mean score for mental functions (IFAP-1_global_) was 1.21 (SD 0.63) and for functional capacities in alternative work (IFAP-2b_global_) 0.87 (SD 0.56). Reliability of IFAP ratings was low to fair (IFAP-1_global_: ICC = 0.46; IFAP-2b_global_: ICC = 0.26), similar to the low interrater reliability of rWC. Agreement showed substantial measurement error: IFAP-1_global_: SEM = 0.47; IFAP-2b_global_: SEM = 0.49. The rWC judgments for claimants with identical ratings in functional limitations (IFAP-2b_global_=1) ranged from 100% to 5%.

**Conclusions:**

Evidence indicates that Functional Assessment, if carried out well, may lead to more reproducibility. This explanatory analysis revealed low to fair interrater reproducibility in mental functions (IFAP-1), in functional capacities (IFAP-2a/b) which extends to rWC. Among various other explanations, we believe this to be mostly due to insufficient training in Functional Assessment and therefore reflects real-world variability in judgment. We recommend revising training format and intensity, and monitoring adherence in practice, followed by re-evaluation of reproducibility of expert judgements. As of today, the outcome is uncertain.

## 1 Introduction

Social security systems provide disability benefits for employees whose work capacity (WC) is noticeably and permanently impaired due to illness or accident. It is common practice for employees claiming disability benefits to undergo an expert evaluation to determine their ability to work (1). What makes expert evaluations a sensitive and controversial issue is the established evidence that different experts reach different conclusions when assessing residual work capacity (rWC) (2). In many countries, including Switzerland, the expert judgment on rWC largely determines the decision on entitlement to disability benefits and their amount.

### 1.1 What motivated our research program

In an illustrative study, 23 psychiatric experts assessed the WC of a hypothetical claimant suffering from recurrent moderate depression (3). Based on identical information (psychiatric history, medical reports, diagnoses from treating physicians, a staged video interview), eight psychiatrists found no impairment, ten concluded partial impairment, and four determined no rWC. The researchers judged such expert variation to be “unacceptable for members of the German state pension system”.

### 1.2 Methodological considerations: high expert variation signals poor reproducibility, crucial in WC assessments

Reproducibility is defined as the degree to which repeated measurements yield similar results (4) and encompasses two distinct concepts, reliability and agreement (4–6). Reliability refers to how well patients and claimants can be distinguished from each other, despite measurement errors, when assessed by two or more raters (interrater reliability), while agreement refers to the absolute differences between expert ratings (interrater agreement) (4). Activities used to perform an evaluation require a high degree of agreement between raters, for example, when evaluating experts as they conduct WC assessments (4, 7). This requirement applies to the evaluation of work (in-)capacity. Of note, high interrater reliability does not imply high interrater agreement (4).

### 1.3 Our solution: develop a structured approach - functional Assessment of WC - to improve expert agreement

A systematic review of 23 studies from 12 countries revealed low to fair reproducibility of experts’ judgments on WC (2). However, disability evaluations that employed a structured approach in both, procedures and outcome measurements, showed higher reproducibility (8). These plausible findings caught our attention. We developed the functional interview, a semi-structured conversation about the claimants’ work, self-perceived work (in-)capacity, and remaining ability to perform work-related tasks and used the social functioning scale Mini-ICF-APP for experts to report their findings. This scale has repeatedly been proposed for assessing work disability in social benefit claims (9–12), although it has not yet been tested in national medicolegal settings. We integrated a scale for mental functions and the Swiss scale for judging rWC related to the last job and alternative work and referred to as “Instruments for Functional Assessment in Psychiatry” (IFAP-1,-2,-3). Following their two-hourly interviews, psychiatrists were instructed to use the IFAPs to document their judgment about the claimants’ functional capacities and limitations in work-related activities (13, 14). This structured approach was named “Functional Assessment” (15).

### 1.4 The RELY-studies showed low expert agreement despite functional assessment and training

Our multicenter reproducibility studies in a real-world setting (RELY-1 and -2) investigated whether the Functional Assessment would improve agreement and reliability for the main outcome, rWC (13). Claimants were assessed by four randomly allocated psychiatrists, all trained in Functional Assessment: one interviewer and three observers who watched the video-taped interview. All experts filled out the IFAP independently from each other. Since RELY-1 showed low agreement and reliability among experts, the study was repeated with more intensive expert training and a much shorter time span between training and application in RELY-2. We have not formally evaluated the effectiveness of the training. In a subsequent comparison, expert agreement for claimants’ rWC in RELY-2 improved by about 20% (reported as standard error of measurement, SEM). Since important decisions for claimants – the entitlement to disability benefits and their amount – were based on these assessments, agreement remained unacceptably low, and measurement error among experts unacceptably high. Interrater reliability of rWC judgments was fair (RELY-1: ICC 0.43; RELY-2: ICC 0.44) and did not change between studies (13).

### 1.5 More work-related conversation with the claimant showed better expert agreement

The content analysis of the RELY-studies investigated the coverage of work-related topics in these interviews to identify factors contributing to the poor reproducibility (16). Prominent finding: Experts asked very little about claimants’ self-perceived activity limitations and work (in-)capacity, which indicated insufficient compliance with the training. Interviews with higher coverage achieved significantly higher expert agreement on WC ratings than those with low coverage, suggesting that interviews conducted with sufficient focus on work may improve reproducibility. Hence, the functional interview on work (15) is a compulsory requirement for completing the IFAP.

### 1.6 Similarities and differences between IFAP scales and mini-ICF-APP scales

The analyses reported in this paper explore the role of the IFAP rating scales used by RELY-experts to quantify mental impairments and capacity limitations observed in medicolegal assessments. IFAP and the social functioning scale Mini-ICF-APP relate to the WHO International Classification of Functioning, ICF and its 5-item-rating scale with generic descriptions (15, 17). The Mini-ICF-APP was developed and validated in occupational rehabilitation (17) and community mental health (18, 19) to evaluate individuals with mental disorders on their functional (in-)capacities in domains of social functioning. The English translation (19) reported high internal consistency (Cronbach’s α 0.869 – 0.912) and good test-retest reliability (ICC 0.832) when applied by two raters who were not described any further (5). The original German study to the Mini-ICF-APP (17, 20) did not publish any psychometric properties. In contrast, the IFAPs are part of the Functional Assessment designed specifically for psychiatric experts who evaluate work (in-)capacity in employees with mental disorders on behalf of social insurers. IFAP takes into account the Swiss medicolegal context, which requires capacity assessment related to the claimants’ last job and to alternative work adjusted to the claimants’ limitations and requires a final judgment on the claimants’ rWC on a scale from 100% to 0%. This judgment should reflect the path from impairments in mental functions (IFAP-1) to limitations in functional capacity (IFAP-2a/-b) to rWC (IFAP-3). Since instruments (here: the Functional Assessment) require validation in the context in which they are being used, IFAP requires validation in the medicolegal context. The ‘reliability studies’ of the Mini-ICF-APP (17–19) include data from controlled research settings of social or rehabilitation context without the purpose to capture real-world heterogeneity. They were reported as classical Spearman rank correlation coefficient (17) which, however, is not a reliability measure (21), or as intraclass correlation coefficients ICCs (18, 19). Classical correlations (e.g., Pearson or Spearman) measure the relationship between two different variables, while the ICC, a true reliability measure, describes the ability to distinguish between subjects within a group (same variable = intra-’class’) (22, 23). It is known that highly correlated observations may have poor agreement (24). Of note, none of the Mini-ICF-APP studies investigated (interrater) agreement (4, 5). The specific procedures (if any), in which the Mini-ICF-APP was used, were not reported in these studies which compromises repeatability. It is legitimate to question whether work disability evaluations under so many “real-world” conditions in a medicolegal context could achieve similarly high interrater reliability if investigated using rigorous methodology (5, 20). This needs to be tested.

### 1.7 Psychiatrists are not work experts

The low agreement in judging rWC observed in RELY possibly reflects psychiatrists’ limited understanding of job demands (2), as psychiatrists are experts in mental health, not work. Hence, mental health professionals might show better agreement on functions which are closer to their mental health expertise, such as the ones reviewed in IFAP-1.

### 1.8 Research objectives

In the RELY-studies, the main outcome was agreement between experts and their reliability when using the medicolegal construct ‘rWC in adjusted work’ (13), derived from the functional assessment (i.e., the IFAP-1 and -2a/b-instruments). Agreement and reliability turned out to be low.

In the current analysis, we examined the functional assessment itself (IFAP 1 and IFAP 2a/b). We hypothesized that the functional assessment should yield better agreement and reliability, given the explicit elaboration of the IFAP 1 and 2a/b items in the manual (15) and their ratings against the specific requirements of adjusted work. Furthermore, we examined the value of the global instruments IFAP-1_global_ and IFAP-2a/b_global_ in predicting rWC in alternative work.

## 2 Methods and material

Reporting of our reliability and agreement studies followed the Guidelines for Reporting Reliability and Agreement Studies, GRRAS-Guidelines (5). The protocol paper describes the key features of the design (14), the main publication reports the findings on the main endpoint rWC (13).

### 2.1 The RELY-studies

We conducted two multicenter reproducibility studies, RELY-1 and -2, with a partial crossover design. Four expert psychiatrists (one interviewer, three video raters) independently rated the rWC of real patients who had claimed disability benefits, four ratings per patient. Protocol (14) and main publication (13) report the details. We recruited 30 claimants for RELY-1 (resulting in 30*4 = 120 ratings) and increased this to 40 claimants for RELY-2. Of these, we recruited 25 new applicants (resulting in 25*4 = 100 ratings) and re-used 15 videos from RELY-1 (resulting in 15*4 = 60 ratings). In total, there were 280 ratings. In the current analysis, we merged the data from RELY-1 and -2 given their high correlations (r=0.88) and did not differentiate between the two different training regimes.

### 2.2 Procedures

The interviews of the claimants conducted in real-world settings were videotaped. Claimants were assessed by four randomly assigned psychiatrists trained in Functional Assessment: one interviewer and three psychiatrists who rated independently the video-taped interview. Pseudo-randomization was used to assign claimants to the interviewer, true randomization to assign them to the observing psychiatrists.

### 2.3 Functional assessments and judgments of work capacity

The three-part IFAP was developed for the RELY-studies (13, 14) to quantify and document impairments in mental functions and limitations in functional domains for assessing rWC, whereby structure and content of IFAP-2 is identical to that of Mini-ICF-APP. IFAP-1 rates twelve mental functions: temperament and personality functions, agreeableness, mental stability, openness to experience, confidence, energy and drive functions, attention functions, memory functions, emotional functions, thought functions, higher-level cognitive functions, experience of self and time functions. IFAP-2 rates functional limitations in thirteen domains: adherence to regulations, planning and structuring of tasks, flexibility, applying expertise, competence to judge and decide, endurance, assertiveness, contact with others, group integration, intimate relationships, non-work activities, self-care, and mobility (15, 25). Both, IFAP-1 and -2, employ the 5-item scale of the ICF rating system: “0”=none, “1”=mild, “2”=moderate, “3”=severe impairment/limitation and “4”=complete disability (26). A rating of “mild” means that the restrictions do not affect WC, while a rating of “moderate” implies that WC is affected (15, 26). IFAP-2a is rated against the profile of the claimants’ last job, IFAP-2b against a profile for alternative work adjusted to the claimants’ limitations. Experts were expected to use the ratings of IFAP-2a/b to judge the claimants’ rWC in the last job (rWC_last_) and in alternative work (rWC_alt_) on a scale from 100% to 0% (IFAP-3a/b). When the experts in RELY-1 had judged a claimant’s rWC_last_ as 100%, they omitted to fill in the IFAP-2b form, which related to alternative work and was initially considered as redundant. This procedure reduced the number of IFAP-2b cases for calculating the ICCs. In RELY-2, this detail was modified and IFAP-2b forms had to be completed regardless of the claimant’s rWC_last_. To facilitate quantitative analyses of the IFAP instruments, we calculated IFAP-1_global_, -2_global,_ -3_global_ scores (“global score”) by taking the mean sum score (= mean across the sum) of the 12 respectively 13 items of the IFAP-1 and -2 scales (17), and the value of the assigned rWC as IFAP-3_global_ score. In this way, we report the summary of the IFAP scales in the same way as the individual domain scales, which facilitates comparison.

### 2.4 Expert certainty in judgments

Expert certainty in their judgment was frequently raised as a potentially strong variable impacting on the variation of rWC. Furthermore, it was postulated that interviewing versus observing experts might vary substantially in their certainty of judgment. To this end, experts were asked to express their certainty in their own rWC judgment (Certainty_rWC_last_ and Certainty_rWC_alt_) on an 11-point Likert scale from 0 (very uncertain) to 10 (very certain). Findings will be reported in **Supplementary 8** only.

### 2.5 Statistics

For question 1, we estimated the interrater reliability of the IFAP ratings using the intraclass correlation coefficient ICC, and the interrater agreement using standard error of measurement SEM and percentage of agreement. We calculated the ICC for the individual IFAP domains (23) and for IFAP_global_ (IFAP-1_global_, -2_global_, -3_global_) taking into account the two-way incomplete crossover design. We fitted a linear mixed-effects model with the IFAP_global_ scores as dependent variable, the intercept as the only fixed effect and psychiatrists and claimants as random effects (using the function “lmer” of the R package “lme4”) (27, 28). Subsequently, we extracted the variance components of the psychiatrists and claimants from the fitted model to calculate the ICC (A, 1). As the judgment from a single rater will be the basis of the measurement, we therefore considered ‘single rater’ type of agreement even though the reliability experiment involved four raters. In summary, the ICC was estimated based on a two-way random-effects model with a single-rating (k = 1), and absolute agreement, using the formula (21):


$$ICC\;\left(A,\;1\right)=\;\frac{\sigma_{cl}^{2}\;}{\sigma_{cl}^{2}+\sigma_{psy}^{2}+\sigma_{cl*psy}^{2}}$$

where cl=claimants, psy=psychiatrists, cl*psy=residuals [claimants*psychiatrist interaction effect and the random measurement error (29)]

The 95% confidence intervals (CI) were obtained by applying a model-based parametric bootstrap for the mixed-effects models with R = 9999 repetitions and reporting the 2.5th and the 97.5th percentiles using the function “bootMer” of the package “lmer” and the function “boot.ci” from the package “boot”. Thus, the calculated ICCs reflect reliability of absolute agreement between raters. We interpreted the ICC as poor (ICC< 0.40), fair (0.40–0.59), good (0.60–0.74) and excellent (> 0.75) (30).

Measurement error for continuous variables is represented by SEM and equals the square root of the error variance. SEM equals the square root of the error variance and is a suitable measure of agreement for continuous variables reported in natural units. It is calculated as $SEM_{agreement}=\sqrt{\sigma_{Psychiatrists}^{2}+\sigma_{Residuals}^{2}\;\;}$ (4, 13). For rWC, SEM is reported as % rWC, while for the unitless IFAP-scale, SEM is a unitless number. Lower SEM values indicate higher agreement because the measurement error is low. Expected value of ‘standard error of measurement’ is defined as SEM_expected_ = $\frac{MAD}{1.96*\;\surd 2}$ (4). If the maximum acceptable difference (MAD) of rWC judgements between raters (psychiatrists and experts) was 25%, the observed ‘standard error of measurement’ had to be smaller than 9.0 percentage points WC [Table 4 in (13)]. Similarly, if we think the maximum acceptable difference of IFAP ratings between raters on a 5-point Likert scale is 1 point, the observed ‘standard error of measurement’ had to be smaller than 0.36 points. For the ordinal data of individual IFAP items (scale from 0 to 4), we focused on clinically relevant disagreement, i.e., impacting rWC. Thus, we created three levels out of the five levels of the IFAP scale: ratings of “no and mild limitations” (mild limitations had been defined as limitations with no impact on WC (15)), “moderate limitations”, and “severe limitations and total disability”. For the 13 single IFAP items regarding rWC_last/alt_, we reported ICC and percentage of agreement based on these 3 levels (4, 5).

For question 2, we analyzed the association between IFAP ratings and judgments on rWC_alt_ (31). First, a scatter plot visualized the relationship between IFAP-2b_global_ versus rWC_alt_. To describe the relationship between the dimensions mental functions, functional capacity and rWC, we divided the range of rWC into 10%-intervals from 100% to 0% and calculated mean and standard deviation of the IFAP-1 and -2a/b values for these intervals separately for rWC estimates in the last job and in alternative work. Each claimant–expert pairing was treated as independent observation (i.e., IFAP ratings were not averaged across the four rating experts). To enable a comparison with previous publications (31, 32), we additional grouped the rWC as high (100% to 70%), medium (69% to 31%) and low (30% to 0%). Second, we performed three univariable linear mixed-effects model (LMM) analyses to examine the effect of IFAP-1_global_, -2a_global_ and -2b_global_ on rWC_alt_, accounting for the potential variability between random effect of claimants and psychiatrist raters. LMMs are particularly suitable for data with hierarchical or nested structures, allowing us to model both fixed effects and random effects. We estimated the model parameters using R lmer (linear mixed-effect model regression) function of the lme4 package, with restricted maximum likelihood (REML) estimation to ensure unbiased estimates of the variance components. Furthermore, we calculated univariable LMM analyses for the 38 individual domains of the three IFAP instruments, which are reported in the supplement.

To test whether the level of expert certainty in his own rWC judgment contributed to the variability (‘low agreement’) of the four experts’ rWC judgments on the same claimant, a linear regression was performed using the absolute deviation of the rWC_last/alt_ of raters from the mean rWC_last/alt_ per claimant as dependent variable and the raters’ certainty in their own rWC_last/alt_ as independent variable. Further, we tested for group differences in certainty in rWC_alt_/rWC_last_ estimation across observer and interviewing raters using independent Welch two sample t-test. Findings will be reported in **Supplementary 8**.

Calculations were carried out in Software R 4.3.2 (2023–10–31) (33). To illustrate expert variation in the interpretation of an IFAP-2b-score of 1 (=mild) and 2 (=moderate) - we fitted a regression line using ordinary least squares, and computed 95% confidence bands using the standard error of the predicted mean response (conducted in R using the ggplot2 and stats packages).

## 3 Results

### 3.1 Participants

Claimants for disability benefits undergoing a psychiatric work disability evaluation at one of four medical assessment centers participated in the study. The mean age of the claimants was 47.8 (SD 9.3) years. All claimants were German-speaking. For claimant characteristics (marital status, nationality, main ICD-10 F-codes) and expert characteristics (age, gender, years of experience, number of work disability evaluations in the year before study participation) see main publication (13).

### 3.2 Reliability and agreement of expert psychiatrists rating mental functions (IFAP-1) and functional capacities (IFAP-2a/b)

#### 3.2.1 Mental functions, IFAP-1, 280 ratings

**Figure 1:**
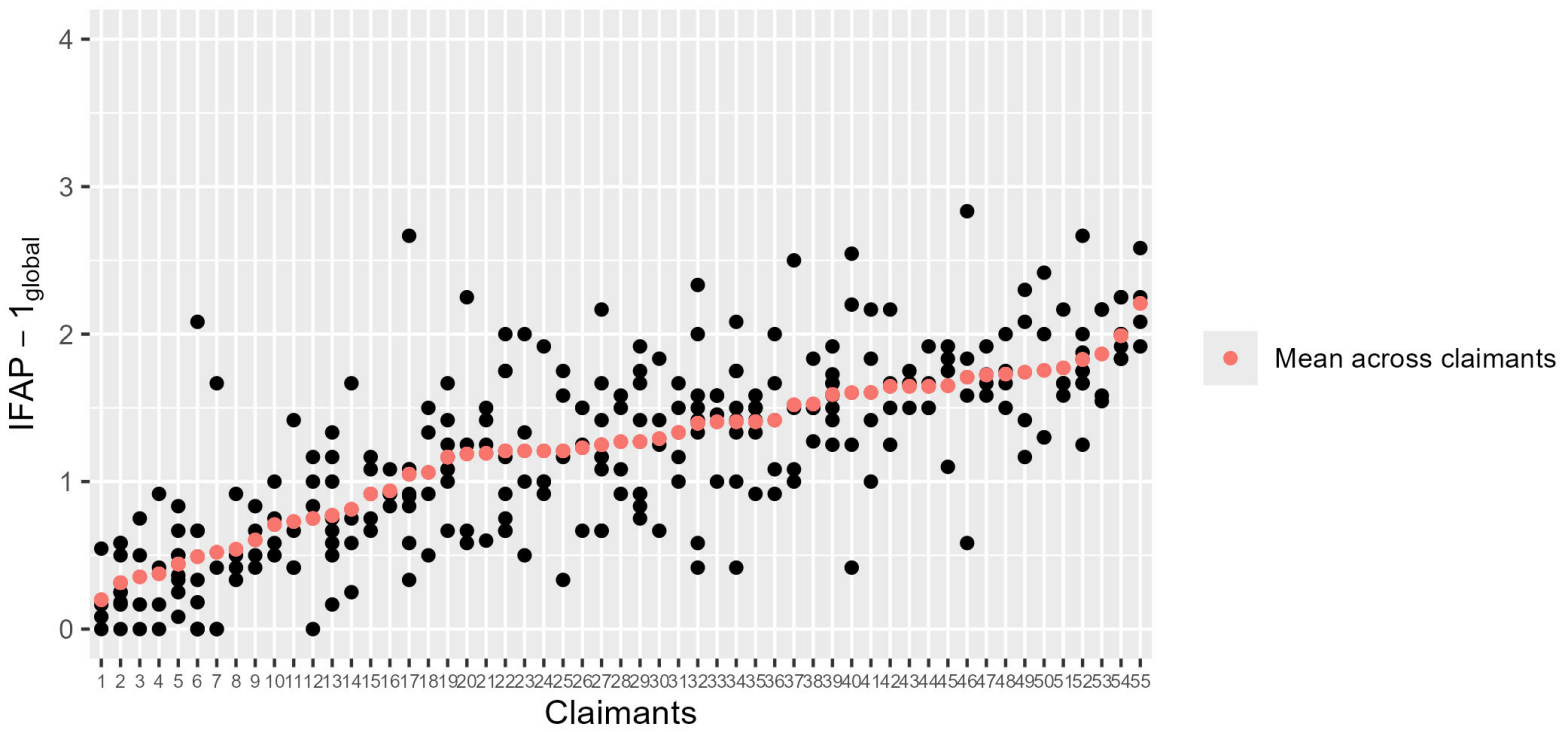
Heterogeneity of the claimant population with regards to impairment in mental functions and variability of the experts rating the impairment of the same claimant. The graph shows to what extent the 55 claimants were impaired in their mental functions (IFAP – 1_global_). Each claimant was rated by 4 experts. 15 claimants from the RELY-1 study were re-rated by different raters in the RELY-2 study (e.g., claimant 5 or 13 or 29 or 39 or 52), resulting in 8 ratings per claimant. The red dots show the mean across the ratings of the 4 (8) experts and are aligned in ascending order. The vertical spread of black dots (= four (eight) individual ratings) illustrates the differences between when rating the same claimant based on the same information. Differences between raters on the same claimant can be substantial. Abbreviations: Instruments of Functional Assessment in Psychiatry, IFAP, with IFAP-1 = Mental Functions; IFAP-2: Functional Capacities; IFAP-3: Swiss scale for rWC. IFAP-2b_global_: mean sum score related to alternative work on a scale from 0 (= no impairment) to 4 (= complete disability).

(**Figure 1**, **Table 1A**, **Supplementary Table S1A, B**). The mean rating of IFAP-1_global_ was 1.21 (SD 0.63). The most impaired mental functions were *Mental Stability* (mean 1.71, SD 0.89), *Self-Confidence (*mean 1.69, SD 0.95), and *Energy* (mean 1.59, SD 0.85). Less than 10% of the individual IFAP-1 ratings indicated severe impairment or complete disability (**Supplementary Table S2**). **Figure 1** illustrates the variation in expert ratings on the same claimant. The reliability of IFAP-1_global_ was 0.46 (ICC, 95% CI 0.32; 0.58) with mainly poor ICC values for the 12 domains ranging from 0.25 to 0.42. Agreement on IFAP-1_global_ among experts measured as SEM was 0.47 (95% CI 0.41; 0.52). Agreement on individual domains, measured in percentage of agreement, was ranging from 54.4% (*Thought Functions*) to 16.4% (*Self Confidence*).

**Figure 2:**
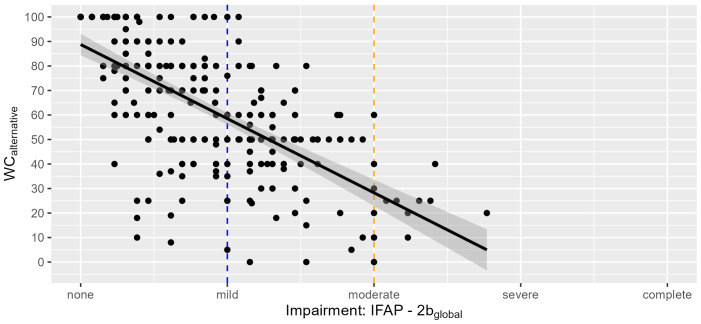
Relationship between the claimants’ functional capacity (IFAP-2b_global_) and their residual work capacity. Each dot represents an expert´s global IFAP-2b rating (scale from 0 to 4) and residual WC_alt_ judgment (alternative work, scale from 0% to 100%; 260 observations). The vertical scatter of dots along the blue line illustrates the variability in WC judgments for the same degree of functional impairment (‘mild’ corresponds to IFAP score of 1.0): Experts judged the claimants’ rWC with mild functional impairment to be between 5% and 100%. For a mean functional impairment of 2.0 (orange line), experts judged the rWC to be between 0% and 60%. The regression line (in black) was fitted using simple univariable regression and is accompanied by its 95% confidence band (in grey). Abbreviations: Instruments of Functional Assessment in Psychiatry, IFAP, with IFAP-1 = Mental Functions; IFAP-2: Functional Capacities; IFAP-3: Swiss scale for rWC. IFAP-2b_global_: mean sum score related to alternative work on a scale from 0 (= no impairment) to 4 (= complete disability).

**Table 1:** Reliability (ICC) and agreement (SEM) of the three IFAP instruments.

The IFAP Instruments	Reliability ICC [95% CI]	Agreement SEM [95% CI]
a) Mental functions		
IFAP-1_global_ (5-item scale)	0.46 [0.32; 0.58]	0.47 [0.41; 0.52]
b) Functional capacities related to the last job		
IFAP-2a_global_ (5-item scale)	0.41 [0.28; 0.53]	0.49 [0.43; 0.54]
Residual Work Capacity_last_ (scale: 0% - 100% rWC)	0.44 [0.3; 0.55]	24.6% rWC [21.9; 27.5]
c) Functional capacities related to alternative work		
IFAP-2b_global_ (5-item scale)	0.26 [0.15; 0.38]	0.49 [0.45; 0.52]
Residual Work Capacity_alt_ (scale: 0% - 100% rWC)	0.45 [0.31; 0.57]	21.49% rWC [19.1; 24.1]

The table reveals the experts' inability to discriminate between claimants based on their functional capacity, IFAP-1_global_, -2a/b_global_ (low ICC), and the low agreement among experts (SEM) in these judgments. Similarly weak findings were observed for the claimants' rWC (IFAP-3a/b) as judged by experts using the functional capacity.

Abbreviations: IFAP, Instrument of Functional Assessment in Psychiatry; IFAP-1, mental functions; IFAP-2a/2b, functional capacities related to last job / alternative work; IFAP-3a/3b, residual work capacity related to last job / alternative work; ICC, Intra-class correlation coefficient; SEM, standard error of measurement.

#### 3.2.2 Functional capacity related to the last job, IFAP-2a, 280 ratings

(**Table 1B** and **Supplementary Table S3A, B**). The mean rating IFAP-2a_global_ was 1.11 (SD 0.64). The domains *Endurance* (mean 2.07, SD 0.81), *Flexibility* (mean 1.56, SD 0.97) and *Assertiveness* (mean 1.44, SD 1.00) revealed the most severe limitations. For all functional domains but *Endurance*, less than 20% of the IFAP-2a ratings indicated severe limitations or complete disability (**Supplementary Table S4**). Reliability of functional capacity ratings for the last job (IFAP-2a_global_) was 0.41 (ICC, 95% CI 0.28; 0.53), with mainly poor ICC values for the 13 domains ranging from 0.20 to 0.43. Agreement among experts on IFAP-2a_global_ was 0.49 (SEM, 95% CI 0.43; 0.54). Percentage of agreement on individual domains was ranging from 82.4% (*Selfcare*) to 15.2% (*Endurance*).

#### 3.2.3 Functional capacity related to alternative work, IFAP-2b, 260 ratings

(**Table 1C** and **Supplementary Tables S5A, B**). The mean rating IFAP-2b_global_ was 0.87 (SD 0.56). Again, the domains *Endurance* (mean 1.68, SD 0.82), *Flexibility* (1.15, SD 0.87), and *Assertiveness* (1.11, SD 0.91) revealed the most severe limitations, although they were rated as less severe compared to IFAP-2a, where the reference was the last job. At all functional domains but *Endurance*, less than 10% of the IFAP-2b ratings indicated severe limitations or complete disability (**Supplementary Table S6**). Reliability of ratings on functional capacity for alternative work, IFAP-2b_global_, was poor (ICC 0.26, 95% CI 0.15; 0.38) as were all ratings on individual IFAP-2b domains. Agreement among experts on IFAP-2b_global_ was 0.49 (SEM, 95% CI 0.45; 0.52). Percentage of agreement on individual domains was ranging from 89.3% (*Selfcare*) to 26.4% (*Endurance*).

In summary, the experts’ interrater reliability for mental functions and functional capacities, both for last job and alternative work (IFAP-1_global_ and -2_global_) was poor to fair. Likewise, the corresponding agreement was poor, too.

### 3.3 Relationship between mental functions, functional capacities, and residual work capacity

Question 2 addressed the association between IFAP-ratings and judgments on rWC in alternative work, using IFAP-2b_global_ and judgments on rWC_alt_ (31) as an example (**Table 2**). The linear mixed-effect regression results showed that the fixed effect of IFAP-2b_global_ explained 38% of the variance in the expert judgment of rWC_alt_.

**Table 2:** Values of the global instruments IFAP-1, IFAP-2a and IFAP-2b in predicting rWC in alternative work.

FAP instruments	Estimate (in % rWC)	Lower 95% CI	Upper 95% CI	Marginal R^2^	n
IFAP-1_global_					
(Intercept)	95.87	88.80	102.92		
IFAP-1_global_	-30.38	-35.04	-25.73	0.45	278
IFAP-2a_global_					
(Intercept)	89.09	82.06	96.10		
IFAP-2a_global_	-27.16	-31.99	-22.38	0.37	260
IFAP-2b_global_					
(Intercept)	86.09	80.06	92.14		
IFAP-2b_global_	-27.32	-32.39	-22.44	0.38	259

Results of the three univariable linear mixed effect regression model in which IFAP-1_global_ and IFAP-2b_global_ were entered separately into the models of rWC_alt_. Marginal R^2^ explains the variance due to fixed effects.

**Figure 2** visualizes the dispersion of rWC judgments among claimants with similar degree of functional limitations: claimants with mild global functional limitations (i.e., an IFAP-2b_global_ value of 1, blue line) were attributed rWCs to be between 5% and 100% in the observed data, between 20% and 97% in the 95% prediction interval (PI) and between 56% and 61% in the 95% confidence interval (CI). Individuals with moderate global functional impairments (i.e., an IFAP-2b value of 2, orange line) were assigned rWCs between 0% and 60% in observed data, between -11% and 67% in the 95% PI and between 23% and 34% in the 95% CI.

The explanatory pathway from ‘mental functions’ to ‘functional abilities’ to ‘rWC’ provides a different perspective. **Table 3** shows the distribution of the IFAP-ratings in 10%-steps rWC for the last job (**Table 3A**) and for alternative work (**Table 3B**). As intended by law, claimants with low and moderate rWC for the last job were attested higher levels of rWC when referred to alternative work adjusted for their functional limitations. The IFAP instruments, however, did not discriminate well between different levels of rWC. This was particularly relevant in the category “moderate rWC” of **Table 3B**. In this category, the law is designed in such a way that even a slight change in rWC impacts the disability benefits.

**Table 3:** Mental functions and functional capacity analyzed by 10% rWC levels.

rWC	100%	90%	80%	70%	60%	50%	40%	30%	20%	10%	0%
a) Last job	High rWC_last_ (n=83)				Moderate rWC_last_ (n=85)			Low rWC_last_ (n=112)			
IFAP-1_global_ (SD) n=280	0.31 (0.36)	0.70 (0.69)	0.74 (0.45)	0.82 (0.35)	1.04 (0.44)	1.34 (0.42)	1.61 (0.40)	1.51 (0.51)	1.46 (0.57)	1.84 (0.42)	1.69 (0.43)
IFAP-2a_global_ (SD) n=280	0.24 (0.20)	0.64 (0.44)	0.63 (0.40)	0.67 (0.37)	0.94 (0.39)	1.16 (0.40)	1.28 (0.40)	1.45 (0.54)	1.29 (0.60)	1.76 (0.44)	1.68 (0.50)
N	30	5	26	22	32	40	13	24	22	12	54
b) Alternative work	High rWC_alt_ (n=128)				Moderate rWC_alt_ (n=99)			Low rWC_alt_ (n=52)			
IFAP-1_global_ (SD) n=279	0.45 (0.42)	0.70 (0.36)	0.93 (0.43)	1.17 (0.54)	1.29 (0.57)	1.47 (0.42)	1.57 (0.38)	1.78 (0.54)	1.81 (0.61)	1.63 (0.38)	1.82 (0.36)
IFAP-2b_global_ (SD) n=260	0.28 (0.30)	0.47 (0.22)	0.60 (0.33)	0.78 (0.36)	1.02 (0.51)	1.14 (0.38)	1.19 (0.48)	1.41 (0.66)	1.53 (0.72)	1.43 (0.74)	1.56 (0.42)
N	44/42*	14	42	28	21	54	24	15	10	7	20/3*

The table shows the mean global score (+ standard deviation) for IFAP-1 and IFAP-2 for each 10% rWC level, separately for rWC judgment in the last job (IFAP-2a top rows) and in alternative work (IFAP-2b bottom rows).

Abbreviations: rWC _last/alt:_ residual work capacity in the last job/in alternative work; IFAP = Instrument of Functional Assessment in Psychiatry. IFAP-1_global_ = global score of impairments in mental functions;IFAP-2a/-2b: global score of functional limitations related to the last job/- to adjusted alternative work; SD: Standard Deviation.

* N sometimes varied due to missing IFAP-2b-ratings. The first value refers to IFAP-1 ratings, the second one to IFAP-2b ratings.

The decline in rWC (from 60% to 50% to 40%) can only marginally be explained by the small decline with large overlapping standard variation in functional abilities of IFAP-2b_alt_ [from 1.02 (mean, SD 0.51) to 1.14 (mean, SD 0.38) to 1.19 (mean, SD 0.48)]. These claimants were quite similar in their level of functioning, and the values do not allow a valid discrimination between adjacent levels of rWC.

In summary, using univariable analysis, IFAP-2b_global_ explains only to a moderate extent the variance in expert judgments of rWC_alt_, the 95% prediction intervals for single patients are quite large, and these ratings provide only limited guidance to expert judgment on rWC_alt_ in individual patients.

## 4 Discussion

### 4.1 Principal findings

This secondary analysis about mental functions (IFAP-1) and functional capacities (IFAP-2) in claimants for work disability was pre-specified to explain the findings of the RELY-studies (14). The assessments with IFAP-1 and IFAP-2 showed low reliability to discriminate between claimants’ functional capacity, i.e. between those with high, moderate, fair, or low capacity. The application of both instruments showed low expert agreement which means that a large ‘measurement error’ in the experts’ assessment led to low agreement between experts when evaluating functions and capacities. These findings resemble the poor to fair reproducibility of expert judgments about rWC in the RELY-studies. Poor reproducibility has already been noted in the experts’ inconsistent assessments of mental functions. It continued in the ratings of functional capacities, and it manifested itself in a wide range of judgments regarding rWC.

### 4.2 Strengths and weaknesses

Strengths: GRRAS guidance states that the interpretation of the results of reliability and agreement studies requires sufficient information on study design and conduct and a good description of the measurement setting and the method of calculation (5). Our design has not been set up to get optimal levels of agreement, rather to reflect real-world performance with all its heterogeneity, and inform insurers, medical and legal professionals and the public. A pre-specified question guided the explanatory analysis of the secondary outcomes (IFAP-1 and -2); the rigorous design of the RELY-studies (13) ensuring trustworthiness in the findings and applicability to the Swiss setting: real WC assessments commissioned by insurers; recruitment of ‘typical’ claimants with a representative spectrum of mental disorders; a large mixed group of psychiatrists; randomly assigned groups of four experts to prevent a rater-group effect, and more (34). Finally, we provide a comprehensive supplement on our data to facilitate comparisons with other studies.

Weaknesses and Limitations: The manual-based training in functional interviewing and defining work demands proved insufficient as was the monitoring of expert compliance to the rating rules prior and during the study. Therefore, we cannot say whether only the training for using the instrument was insufficient or whether the instrument did not work as expected. RELY-1 suffered a serious setback when changes in the governmental administration led to a one-year disruption resulting in a change of the research design (13).

### 4.3 Methodological considerations related to the low reproducibility

#### 4.3.1 Rating the IFAP - User training and compliance

The innovative component of RELY is the Functional Assessment as described in the introduction (13, 14, 16) and in **Supplementary S7**. Manual-based training should ensure that experts stick to the semi-structured interview and all apply the same criteria in their rating judgments. Psychiatrists were told to rate the claimants’ limitations to a reference, “the claimant’s last job” (IFAP-2a) or to “suitable alternative work adjusted to the limitations” (IFAP-2b). Informative job descriptions for suitable alternative work (‘hotel jobs’) were provided as part of our study. While claimants have varying capacity profiles with regards to the limitations, the reference was always the “job requirements” and their match with the “claimants’ (in-) capacities”. If a claimant had a severe agoraphobia, but was only working in home office, this severe limitation had no impact on his job.

The content analysis of RELY-1 documents poor compliance with the two most important steps of the functional interview – the enquiry of self-perceived work limitations and of work-related health complaints [median number of enquiries: 0 to 1.5 coding units (= smallest meaningful unit of a text)] (16): The relationship between the claimants’ functional capacity in adjusted work (IFAP-2b_global_) and the rWC assigned by the expert in **Figure 2** highlights an example how experts did not follow guidance in translating capacity limitations into the final judgment of WC: The rule for “mild impairment” stipulated that the claimants’ limitations do not affect WC, while functional limitations that affect WC need to be rated as “moderate limitations”. Nevertheless, experts assigned claimants with mild functional limitations a rWC of between 5% and 100%. If they had adhered to the RELY-framework, most dots to the left of the blue line would have to lie at 100% WC. Dots below 100% indicate user errors. When experts assign work incapacities if the mean global rating of IFAP-2b is 1 or below, they failed to understand the IFAP rating.

Others argued that - for instance - a rating of 3 (severe impairment) in two domains and all other domains being 0, would have resulted in a mean sum score of (6/13 == 0.46). Nevertheless, such a person might experience severe limitations to work. This theoretical constellation, however, was very rarely seen in practice, if at all. If claimants had substantial impairments (e.g., score of 3) in one or two domains, almost all mild to moderate impairments in others which shifted their total score above a mean sum score of 1. [Personal communication with co-author J. Jeger who collected mini-ICF-ratings from more than 1000 claimants over a 10-year period (31)].

**Figure 2** shows, above all, that we did not succeed in enforcing our own guidance in the study. We recognise insufficient user training as one of the biggest problems with both, RELY-1 and -2.

#### 4.3.2 Reliability and agreement in IFAP-ratings

High interrater reliability indicates that two or more experts can well distinguish between claimants with high, moderate, low and very low functional capacity. Low reliability means that experts are unable to discriminate claimants. Apart from insufficient training (misclassification, inconsistent application of rules, disagreement in judgments), poor interrater reliability can occur when instruments, like the 5-point IFAP scale, have only few levels to describe claimants’ functioning when their level of functional limitations varies considerably. Some suggest that scales with 7 to 10 points are best for achieving valid reliability (35, 36). This, however, presupposes that the specific scale values can be precisely defined and operationalized, and that psychiatrists can differentiate the functional limitations accordingly. The authors of this paper expressed strong doubts that these preconditions can be met.

#### 4.3.3 Agreement

Agreement informs about the “measurement error” of an instrument. It becomes low when the measurement error exceeds patient variance. “Our instrument” for the Functional Assessment was an expert with a range of competencies: trained in collecting relevant information from claimants, experienced in using appropriate instruments (e.g., tests validated in similar settings to the one in which they were used), with a good understanding of work requirements, and the ability to transform the information collected into reasoned judgment about functional capacities for work. These were high expectations. The co-authors of this paper concluded that inadequate training and a lack of supervising the use of the instrument were the most probable reasons for the disagreement (5).

#### 4.3.4 Claimants and settings

Since a person’s level of functioning is an interaction between her or his health conditions and environmental factors (ICF) (26), claimants need to be evaluated against an explicit reference setting (e.g., last vocational setting, general working life, general labor market), which will affect the level of disability in that specific setting (26). This requirement is also justified from a methodological perspective, as reliability and agreement coefficients are population- and context-specific (5). To this end, our study referenced the evaluation to the claimants’ last job and an alternative work with explicit description of the main functional demands. Such information is crucial to allow comparison of findings across studies, but it is often not or not adequately reported [e.g. ‘uniform standard environment’ (18)]. Taken alone, numerical values of ratings or reproducibility measures without context have limited meaning and hinder cross-study comparisons.

#### 4.3.5 The impact of real-world raters

The RELY-studies were designed to mimic the diversity of real-world assessments (2): Experts vary in their professional approach (e.g., behavioral therapy, systemic therapy, psychoanalysis), in their setting (hospital, community centers, individual practice, rehabilitation, forensic), in their experience in performing medical evaluations. The setting determines the kind of patients they see, experience determines judgments (2, 21, 37). These features strengthen our conclusion that the observed low interrater reliability and agreement on functioning and WC reflects the real world of the Swiss setting.

#### 4.3.6 Talking about work works

To align professional heterogeneity, we had trained the experts in collecting work-related information from claimants using a semi-standardized five-step interview about claimants’ perceptions of their work and functional limitations (38). While our content analysis of the RELY-1 interviews revealed that compliance with the training had been low (16), groups with interviewers who did comply, achieved significantly higher agreement in their rWC judgments. This confirms the need for more training, for checking the learning success and for monitoring its use in practice.

### 4.4 Comparison to other studies

Overall, we noticed a lack of research for comparison. We identified three studies in patients with mental disorders (17–19) on the reproducibility of the global score of the Mini-ICF-APP (the instrument underlying IFAP-2). None of them was carried out as part of a medicolegal assessment with the aim of determining the applicants’ ability to work and to serve as a basis for a decision on a disability pension. One study investigated inpatients in a psychosomatic rehabilitation clinic (17), two other studies took place in community mental health centers in Italy (18) and the UK (19). Reliability and agreement are not fixed properties of instruments, rather, they are the product of interactions between purpose, subjects or objects, instruments, setting, conduct and analysis (5). Since these studies differ in important ways from our medicolegal study, a direct comparison is not informative.

### 4.5 Comparing the RELY-data with findings from routine care

Important insights can be gained from a comparison with a recent single-center study (ScS) about Mini-ICF-APP assessments on more than 900 consecutive claimants (31). Similarities between the two studies include the medicolegal context, claimants randomly commissioned from the same national insurer, and rWC_alt_ as outcome. Studies differed in that RELY-claimants participated voluntarily while ScS-claimants underwent a routine assessment with Mini-ICF-APP ratings. RELY included 40 distinct psychiatrists, while three freelance ScS-psychiatrists assessed 84% of claimants over 10 years. The ScS did not investigate reproducibility. Finally, the ScS seems to allow very different procedures of assessment in which the Mini-ICF is applied.

The ScS found higher functional limitations [ScS: Mini-ICF_global_ 1.39 (mean, SD 0.60) vs. RELY: IFAP-2b_global_ 0.87 (mean; SD 0.56)] and lower rWC_alt_ [ScS: 50.6% (mean, 95% CI 48.7; 52.5) vs. RELY: 59.2% (mean, 95% CI 55.7; 62.6)]. Apart from differences in design, the recruitment of ScS-claimants included those with more functional limitations, while the voluntary participation in RELY may have attracted claimants with less severe limitations. Alternatively, ScS-experts may have been more lenient and attributed higher levels of limitations and consequently lower rWC than the more representative mix of RELY-experts. Both explanations suggest possible bias highlighting the need for integrating methodological procedures to protect against biased selection of claimants and experts in future studies.

### 4.6 Implications for the practice of work disability assessments and further research

WC evaluations require an in-depth exchange about work between claimant and expert. Our content analysis of RELY-1 showed that this in-depth exchange did not take place (16). However, many psychiatrists do not see themselves as experts on work and work demands. In a representative survey, experts from various disciplines expressed their need for tools when assessing WC. Their expectations: high predictiveness, high interrater agreement, and comprehensive for laypeople (39). Functional interviewing (15, 16) has a strong face validity and could serve as a framework. Regrettably, the RELY-studies did not deliver “proof of concept”. The main reasons identified were internal factors (e.g., training insufficient to change behavior) and experts’ non-compliance with the procedure. Both are modifiable. Increased training in RELY-2 led to better coverage of the topics of the functional interview (manuscript finalized). This justifies a second effort to validate the concept: Adjust training, ensure that raters know the rating rules and apply them accurately, monitor learning progress and compliance in practice. This validation approach should determine the impact of functional interviewing on expert agreement and reliability about work (in-)capacity (16). As of today, the outcome is uncertain. If positive, follow-up studies could investigate the impact of innovative schemes like online training programs or calibration sessions on reducing variability and improving the practical use of IFAP in social security contexts.

The framework could be complemented by additional psychometric tools on functional diagnostics and prognostics: Digitally Assisted Standard Diagnostics in Insurance Medicine (DASDIM) for mental disorders (40) or the Work Disability–Functional Assessment Battery (WD-FAB) for physical and behavioral functions (41), which is currently translated into German (42) and French (43). Such instruments, validated in the medicolegal context can facilitate consistency checks about the claimants’ self-perceived capacity limitations and thereby contribute to evidence-based decisions.

Some may argue, why bother with tools that do not live up to expectations? First, the most plausible factors as to why the IFAP rating did not work as expected can be modified. Second, the Mini-ICF-APP (underlying the IFAP) is currently in place in multiple settings, such as expert training (SIM, www.swiss-insurance-medicine.ch), psychiatric assessments and as guidance for psychiatric assessments (11). Third, the Functional Assessment and IFAP-instruments are the only fully evaluated instruments developed in the national setting. If further research shows that they do not work as required, they should no longer be used, and their flawed results should not be employed to determine disability benefits. The alternative, starting from scratch in search of a better tool, is time-consuming and resource-intensive with an uncertain ending.

Not acting is not an option. Work disability assessments for decision-making on granting benefits are subject to the societal legal principle “Equality before the law”: People with similar level of limitations in similar work settings should be treated equally. Fulfilling this principle expects experts to reliably distinguish between people with high, moderate, fair and low ability to work (“reliability”), and to achieve a higher level of agreement with other experts in their decisions. This principle is currently under scrutiny. If no progress can be made in the current allocation of disability benefits based on functional impairments of WC, policymakers may need to consider changes to the framework to ensure equal treatment. This may include a change in the law.

## 5 Conclusions

Integrating the findings of the IFAP-analyses with the findings of other RELY-analyses, we conclude that Functional Assessment if carried out well, can lead to more reproducibility (16). This explanatory analysis of the RELY-data revealed low to fair interrater reproducibility for mental functions (IFAP-1) for functional capacities (IFAP-2a/b) and finally for rWC. Among various other explanations, we think this to be mostly due to insufficient training in Functional Assessment. Conducting work disability assessments as currently taught and practiced is not likely to improve the poor reliability and the poor agreement, regardless of the instrument used. Rather than starting from scratch in search of a better tool, we recommend revising training format, delivery and intensity, and monitor adherence in routine practice, followed by re-evaluation of reproducibility of expert judgments. As of today, the outcome is uncertain.

## Data Availability

The data analyzed in this study is subject to the following licenses/restrictions: The raw data supporting the conclusions of this article will be made available quickly and easily by the authors, on condition that the researchers are at a reputable academic institution and that they accept the conditions of use. Requests to access these datasets should be directed to Regina Kunz regina.kunz@usb.ch.

## References

[1] Baumberg Geiger B Garthwaite K Warren J Bambra C . Assessing Work Disability for Social Security Benefits: International Models for the Direct Assessment of Work Capacity. Disabil Rehabil. (2018) 40(24):2962–70. doi: 10.1080/09638288.2017.1366556, PMID: 28841811

[2] Barth J de Boer WE Busse JW Hoving JL Kedzia S Couban R . Inter-rater agreement in evaluation of disability: systematic review of reproducibility studies. BMJ. (2017) 356:j14. doi: 10.1136/bmj.j14, PMID: 28122727 PMC5283380

[3] Dickmann JR Broocks A . Psychiatric expert opinion in case of early retirement–how reliable]? Fortschr Neurol Psychiatr. (2007) 75:397–401. doi: 10.1055/s-2006-944303, PMID: 17031778

[4] de Vet HC Terwee CB Knol DL Bouter LM . When to use agreement versus reliability measures. J Clin Epidemiol. (2006) 59:1033–9. doi: 10.1016/j.jclinepi.2005.10.015, PMID: 16980142

[5] Kottner J Audige L Brorson S Donner A Gajewski BJ Hrobjartsson A . Guidelines for reporting reliability and agreement studies (GRRAS) were proposed. J Clin Epidemiol. (2011) 64:96–106. doi: 10.1016/j.jclinepi.2010.03.002, PMID: 21130355

[6] Tinsley HEA Weiss DJ . Interrater reliability and agreement. In: Handbook of applied multivariate statistics and mathematical modeling. Academic Press, San Diego (2000). p. 95–124.

[7] Guyatt G Walter S Norman G . Measuring change over time: assessing the usefulness of evaluative instruments. J Chronic Dis. (1987) 40:171–8. doi: 10.1016/0021-9681(87)90069-5, PMID: 3818871

[8] Spanjer J Krol B Brouwer S Groothoff JW . Inter-rater reliability in disability assessment based on a semi-structured interview report. Disabil Rehabil. (2008) 30:1885–90. doi: 10.1080/09638280701688185, PMID: 19037781

[9] Ebner G Dittmann V Mager R Stieglitz R-D Träbert S Bührlen B . Final report: Development of guidelines for the assessment of mental disabilities. In: The formal quality of psychiatric assessment reports. Basel Universitäre Psychiatrische Kliniken, UPK (2011).

[10] Colomb E Dittmann V Ebner G Hermelink U Hoffmann-Richter U Kopp HG . Qualitätsleitlinien für psychiatrische Gutachten in der Eidgenössischen Invalidenversicherung. Steinhausen: Swiss Society of Psychiatry and Psychotherapy (2012).

[11] Ebner G Colomb E Mager R Marelli R Rota F . Quality guidelines for insurer reports on psychiatric assessments (2016). Bern: Swiss Society of Psychiatry and Psychotherapy [SGPP]. Available online at: https://www.psychiatrie.ch/sgpp/fachleute-und-kommissionen/leitlinien (Accessed November 4, 2025).

[12] Riemer-Kafka G . Medical expertises for insurers. An interdisciplinary guidance on medical and legal issues. 2nd ed. AG Bern: Universität Luzern: Stämpfli Verlag (2012).

[13] Kunz R von Allmen DY Marelli R Hoffmann-Richter U Jeger J Mager R . The reproducibility of psychiatric evaluations of work disability: two reliability and agreement studies. BMC Psychiatry. (2019) 19:205. doi: 10.1186/s12888-019-2171-y, PMID: 31266488 PMC6607597

[14] Bachmann M de Boer W Schandelmaier S Leibold A Marelli R Jeger J . Use of a structured functional evaluation process for independent medical evaluations of claimants presenting with disabling mental illness: rationale and design for a multi-center reliability study. BMC Psychiatry. (2016) 16:271. doi: 10.1186/s12888-016-0967-6, PMID: 27474008 PMC4966817

[15] de Boer W Marelli R Hoffmann-Richter U Eichhorn M Jeger J Colomb E . Functional assessment in psychiatry. A manual. [Die funktionsorientierte begutachtung in der psychiatrie. Basel: Research & Education, University of Basel (2015).

[16] von Allmen DY Kedzia S Dettwiler R Vogel N Kunz R de Boer WEL . Functional interviewing was associated with improved agreement among expert psychiatrists in estimating claimant work capacity: A secondary data analysis of real-life work disability evaluations. Front Psychiatry. (2020) 11:621. doi: 10.3389/fpsyt.2020.00621, PMID: 32719624 PMC7350701

[17] Linden M Baron S Muschalla B . Mini-ICF-APP. Mini-ICF-Rating für Aktivitäts- und Partizipationsbeeinträchtigungen bei psychischen Erkrankungen. 2 ed. Bern: Hogrefe (2015).

[18] Balestrieri M Isola M Bonn R Tam T Vio A Linden M . Validation of the Italian version of Mini-ICF-APP, a short instrument for rating activity and participation restrictions in psychiatric disorders. Epidemiol Psychiatr Sci. (2013) 22:81–91. doi: 10.1017/S2045796012000480, PMID: 22989494 PMC6998349

[19] Molodynski A Linden M Juckel G Yeeles K Anderson C Vazquez-Montes M . The reliability, validity, and applicability of an English language version of the Mini-ICF-APP. Soc Psychiatry Psychiatr Epidemiol. (2013) 48:1347–54. doi: 10.1007/s00127-012-0604-8, PMID: 23080483

[20] Baron S Linden M . Disorders of functions and disorders of capacity in relation to sick leave in mental disorders. Int J Soc Psychiatry. (2009) 55:57–63. doi: 10.1177/0020764008091660, PMID: 19129326

[21] Streiner DL Norman GR Cairney J . Health measurement scales. A practical guide to their development and use. 5th ed. Oxford: Oxford University Press (2015).

[22] Karanicolas PJ Bhandari M Kreder H Moroni A Richardson M Walter SD . Evaluating agreement: conducting a reliability study. J Bone Joint Surg Am volume. (2009) 3:99–106. doi: 10.2106/JBJS.H.01624, PMID: 19411507

[23] Hernaez R . Reliability and agreement studies: a guide for clinical investigators. Gut. (2015) 64:1018–27. doi: 10.1136/gutjnl-2014-308619, PMID: 25873640

[24] Ranganathan P Pramesh CS Aggarwal R . Common pitfalls in statistical analysis: Measures of agreement. Perspect Clin Res. (2017) 8:187–91. doi: 10.4103/picr.PICR_123_17, PMID: 29109937 PMC5654219

[25] Linden M Baron S Muschalla B Ostholt-Corsten M . Fähigkeitsbeeinträchtigungen bei psychischen Erkrankungen. Diagnostik, Therapie und sozialmedizinische Beurteilung in Anlehnung an das Mini-ICF-APP. Bern: Huber (2015).

[26] World Health Organisation . International classification of functioning (2001). Disability and Health. Available online at: http://www.who.int/classifications/icf/en/ (Accessed February 12, 2025).

[27] McGraw K Wong S . Forming inferences about some intraclass correlation coefficient. psychol Methods. (1996) 1:30–46. doi: 10.1037/1082-989X.1.1.30

[28] Koo TK Li MY . A guideline of selecting and reporting intraclass correlation coefficients for reliability research. J chiropractic Med. (2016) 15:155–63. doi: 10.1016/j.jcm.2016.02.012, PMID: 27330520 PMC4913118

[29] Shrout PE Fleiss JL . Intraclass correlations: uses in assessing rater reliability. psychol bulletin. (1979) 86:420–8. doi: 10.1037/0033-2909.86.2.420, PMID: 18839484

[30] Fleiss JL . Statistical methods for rates and proportions. 2 ed. New York: Wiley (1981).

[31] Rosburg T Kunz R Trezzini B Schwegler U Jeger J . The assessment of capacity limitations in psychiatric work disability evaluations by the social functioning scale Mini-ICF-APP. BMC Psychiatry. (2021) 21:480. doi: 10.1186/s12888-021-03467-w, PMID: 34592979 PMC8485557

[32] Jeger J Trezzini B Schwegler U . Applying the ICF in disability evaluation: a report based on clinical experience. In: Escorpizo R Brage S Homa D Stucki G , editors. Handbook of vocational rehabilitation and disability evaluation: Application and implementation of the ICF. Springer, Cham (2015). p. 397–410.

[33] R Core Team . R: A language and environment for statistical computing. R Foundation for Statistical Computing (2018). Available online at: https://www.r-project.org/ (Accessed December 04, 2025).

[34] Crits-Christoph P Johnson J Gallop R Gibbons MB Ring-Kurtz S Hamilton JL . A generalizability theory analysis of group process ratings in the treatment of cocaine dependence. Psychother research: J Soc Psychother Res. (2011) 21:252–66. doi: 10.1080/10503307.2010.551429, PMID: 21409739 PMC3361025

[35] Scherpenzeel A . Why use 11-point scales. Lausanne: Swiss Center of Social Sciences in Lausanne (2018). Available online at: https://forscenter.ch/wp-content/uploads/2018/10/varia_11pointscales.pdf (Accessed December 04, 2025).

[36] Scherpenzeel AC . A question of quality: evaluating survey questions by multi trait - multi method studies. Amsterdam: University of Amsterdam (1995).

[37] Kobak KA Brown B Sharp I Levy-Mack H Wells K Ockun F . Sources of unreliability in depression ratings. J Clin psychopharmacology. (2009) 29:82–5. doi: 10.1097/JCP.0b013e318192e4d7, PMID: 19142114

[38] Anner J Kunz R de Boer W . Reporting about disability evaluation in European countries. Disabil Rehabil. (2014) 36:848–54. doi: 10.3109/09638288.2013.821180, PMID: 23919642

[39] Schleifer R Gamma A Warnke I Jabat M Rossler W Liebrenz M . Online survey of medical and psychological professionals on structured instruments for the assessment of work ability in psychiatric patients. Front Psychiatry. (2018) 9:453. doi: 10.3389/fpsyt.2018.00453, PMID: 30319460 PMC6167551

[40] Rosburg T Deuring G Ebner G Hauch V Pflueger MO Stieglitz RD . Digitally Assisted Standard Diagnostics in Insurance Medicine (DASDIM): psychometric data in psychiatric work disability evaluations. Disabil Rehabil. (2023) 45:4457–70. doi: 10.1080/09638288.2022.2151655, PMID: 36523117

[41] Marfeo EE Ni P McDonough C Peterik K Marino M Meterko M . Improving assessment of work related mental health function using the work disability functional assessment battery (WD-FAB). J Occup rehabilitation. (2018) 28:190–9. doi: 10.1007/s10926-017-9710-5, PMID: 28477069 PMC8935348

[42] Weinbrenner S . Adaptation and validation of the Work Disability Functional Assessment Battery (WD-FAB) to German. German Pension Fund. EUMASS congress: Insurance Medicine 2.0 in a Changing World. Strasbourg. (2023).

[43] Vermein E van Damme S . Psychometric validation of the work disability - Functional Assessment Battery (WD-FAB) for Belgium. 2023-2026. INAMI Institut national d’assurance maladie-invalidité Ghent University . Ghent Health Psychology Lab. Available online at: https://research.ugent.be/web/result/project/1a573cf3-fbe6-4584-9a18-a69859e78f5d/details/160a00123-psychometric-validation-of-the-work-disability—functional-assessment-battery-wd-fab-for-belgium/en (Accessed December 4, 2025).

