# Supplementary material for: The reproducibility of structured functional assessments in a social security setting: a pre-specified explanatory analysis of the RELY-studies
Source: Front Psychiatry. 2026 Jan 2;16:1643221. doi: 10.3389/fpsyt.2025.1643221 (PMC12808417; doi:10.3389/fpsyt.2025.1643221)
Supplement: Supplementary file 1 [file DataSheet1.docx]

Supplementary materials

**The reproducibility of structured functional assessments in a social security setting. A pre-specified explanatory analysis of the RELY-studies**

Regina Kunz et al.

**Affiliation**

University of Basel Hospital, Department of Clinical Research, EbIM Research & Education, Basel, Switzerland

***Corresponding author:**

E-mail: [regina.kunz@usb.ch](mailto:regina.kunz@usb.ch)

**Table S1:** **Mental functions of the IFAP instrument (IFAP-1): Individual domains**

1. Ratings, means and standard deviation

| IFAP-1 | N | Mean | SD |
| --- | --- | --- | --- |
| (1) functions of temperament and personality | 277 | 1.53 | 0.87 |
| (2) agreeableness | 279 | 0.83 | 0.78 |
| (3) mental stability | 279 | 1.71 | 0.89 |
| (4) openness towards new experiences | 269 | 1.12 | 0.95 |
| (5) self confidence | 277 | 1.69 | 0.95 |
| (6) energy | 279 | 1.59 | 0.85 |
| (7) attention | 278 | 1.09 | 0.91 |
| (8) memory | 276 | 0.84 | 0.85 |
| (9) emotional functions | 276 | 1.53 | 0.89 |
| (10) thought functions | 278 | 0.78 | 0.83 |
| (11) higher-level cognition | 275 | 0.99 | 0.97 |
| (12) experience of self and of time | 274 | 0.83 | 0.91 |
|  |  |  |  |
| IFAP-1_global_ | 280 | 1.21 | 0.63 |

1. Reliability and agreement

| IFAP-1 | ICC [95% CI] | Percentage of Agreement  (in %) based on 3 categories |
| --- | --- | --- |
| (1) temperament and personality | 0.36 [0.23, 0.49] | 23.88 |
| (2) agreeableness | 0.36 [0.23, 0.48] | 53.62 |
| (3) mental stability | 0.39 [0.25, 0.51] | 20.29 |
| (4) openness towards new experiences | 0.27 [0.14, 0.39] | 30.00 |
| (5) self confidence | 0.42 [0.28, 0.54] | 16.42 |
| (6) energy | 0.31 [0.18, 0.43] | 24.64 |
| (7) attention | 0.30 [0.17, 0.42] | 36.76 |
| (8) memory | 0.25 [0.14, 0.37] | 43.28 |
| (9) emotional functions | 0.34 [0.21, 0.46] | 24.24 |
| (10) thought functions | 0.30 [0.17, 0.42] | 54.41 |
| (11) higher-level cognition | 0.36 [0.23, 0.48] | 32.31 |
| (12) experience of self and of time | 0.28 [0.16, 0.4] | 44.62 |
|  |  |  |
| Reliability and Agreement of IFAP-1_global_ | **ICC [95% CI]** | **SEM [95% CI]** |
| IFAP-1_global_ | 0.46 [0.32, 0.58] | 0.47 [0.41, 0.52] |

This table shows the reliability (intraclass correlation coefficient, ICC) and agreement (expressed as percentage of agreement between four raters) for the twelve IFAP-1 domains and for the IFAP-1_global_ score (expressed as standard error of measurement, SEM). Since we were interested in the IFAP categories that impacted on work capacity, we pooled the levels (0+1) and (3+4) to calculate percentage of agreement. The mean resp. median of the 12 IFAP domains was 33.7% resp. 31.2% agreement. Both, ICCs and percentage of agreement of the individual domains, achieved only low values, while reliability and agreement (SEM) based on the global score achieved fair values.

**Table S2: Distribution of ratings on mental functions (IFAP-1)**

|  | “0”  No  impairment | “1”  Mild  impairment | “2”  Moderate impairment | “3”  Severe impairment | “4”  Complete  disability |
| --- | --- | --- | --- | --- | --- |
| (1) functions of temperament and personality | 38  13.7% | 86  31.0% | 120  43.3% | 33  11.9% | 0  0.0% |
| (2) agreeableness | 108  38.7% | 115  41.2% | 51  18.3% | 5  1.8% | 0  0.0% |
| (3) mental stability | 29  10.4% | 74  26.5% | 126  45.2% | 49  17.6% | 1  0.4% |
| (4) openness towards new experiences | 88  32.7% | 79  29.4% | 83  30.9% | 19  7.1% | 0  0.0% |
| (5) confidence | 40  14.4% | 61  22.0% | 121  43.7% | 55  19.9% | 0  0.0% |
| (6) energy | 32  11.5% | 86  30.8% | 126  45.2% | 35  12.5% | 0  0.0% |
| (7) attention | 88  31.7% | 93  33.5% | 82  29.5% | 15  5.4% | 0  0.0% |
| (8) memory | 115  41.7% | 98  35.5% | 54  19.6% | 9  3.3% | 0  0.0% |
| (9) emotional functions | 42  15.2% | 79  28.6% | 122  44.2% | 33  12.0% | 0  0.0% |
| (10) thought functions | 125  45.0% | 95  34.2% | 51  18.3% | 7  2.5% | 0  0.0% |
| (11) higher-level cognition | 112  40.7% | 72  26.2% | 73  26.5% | 18  6.5% | 0  0.0% |
| (12) experience of self and of time | 127  46.4% | 79  28.8% | 55  20.1% | 13  4.7% | 0  0.0% |
|  |  |  |  |  |  |
| Total ratings per category (n=3317) | 944 | 1017 | 1064 | 291 | 1 |
| Distribution (%) | 28% | 31% | 32% | 9% | 0% |

**Table S3: Functional impairments related to last job (IFAP-2a): Individual domains**

1. Ratings, mean, standard deviation

|  | N | Mean | SD |
| --- | --- | --- | --- |
| (1) adherence to regulations | 278 | 0.92 | 0.96 |
| (2) planning and structuring of tasks | 275 | 1.16 | 1.08 |
| (3) flexibility | 279 | 1.56 | 0.97 |
| (4) applying expertise | 271 | 0.86 | 0.98 |
| (5) competence to judge and decide | 276 | 1.06 | 1.03 |
| (6) endurance | 275 | 2.07 | 0.81 |
| (7) assertiveness | 275 | 1.44 | 1.00 |
| (8) contact with others | 275 | 1.07 | 0.91 |
| (9) group integration | 268 | 1.14 | 0.97 |
| (10) intimate relationships | 272 | 0.62 | 0.86 |
| (11) non-work activities | 272 | 0.85 | 0.89 |
| (12) self care | 276 | 0.80 | 0.92 |
| (13) mobility | 274 | 0.78 | 0.87 |
|  |  |  |  |
| IFAP-2a_global_ | 280 | 1.11 | 0.64 |

.

1. Reliability (intraclass correlation coefficient, ICC) and agreement (percentage of agreement and standard error of measurement, SEM) between four raters

| IFAP-2a | ICC [95% CI] | Percentage of Agreement  (in %) based on 3 categories |
| --- | --- | --- |
| (1) regulations | 0.22 [0.11, 0.34] | 36.76 |
| (2) planning | 0.26 [0.14, 0.38] | 29.23 |
| (3) flexibility | 0.32 [0.19, 0.45] | 21.74 |
| (4) applying expertise | 0.28 [0.16, 0.40] | 52.46 |
| (5) competence to judge | 0.34 [0.21, 0.46] | 37.88 |
| (6) endurance | 0.26 [0.13, 0.38] | 15.15 |
| (7) assertiveness | 0.30 [0.17, 0.42] | 18.46 |
| (8) contact with others | 0.30 [0.17, 0.42] | 31.82 |
| (9) group integration | 0.31 [0.18, 0.43] | 28.81 |
| (10) intimate relationships | 0.43 [0.29, 0.55] | 39.66 |
| (11) non-work activities | 0.26 [0.13, 0.39] | 22.58 |
| (12) self-care | 0.20 [0.09, 0.31] | 82.35 |
| (13) mobility | 0.21 [0.10, 0.32] | 65.15 |
|  |  |  |
| Reliability and Agreement of IFAP-2a_global_ | **ICC [95% CI]** | **SEM [95% CI]** |
| **IFAP-2a_global_** | 0.41 [0.28, 0.53] | 0.49 [0.43, 0.54] |
| **rWC_last_ [%]** | 0.44 [0.3, 0.55] | 24.65 [21.93, 27.49] |

This table shows the reliability (ICC) and agreement (percentage of agreement between four raters) for the thirteen IFAP-2a domains and for the IFAP-2a_global_ score (agreement expressed as SEM). Since we were interested in the IFAP categories that impacted on work capacity, we pooled the IFAP levels (0+1) and (3+4) to calculate percentage of agreement. The mean resp. median of the 13 IFAP-2a domains was 37.08% resp. 31.82% agreement. Both, ICCs and percentage of agreement of the individual domains, achieved only low values. The global measures, IFAP-2a_global_  and WC_last_ reached poor to fair reliability values, while agreement (SEM) was poor in both parameters.

**Table S4: Distribution of ratings on functional impairment related to the last job (IFAP-2a)**

|  | “0”  No  impairment | “1”  Mild  impairment | “2”  Moderate impairment | “3”  Severe impairment | “4”  Complete disability |
| --- | --- | --- | --- | --- | --- |
| (1) adherence to regulations | 116  41.7% | 90  32.4% | 50  18.0% | 21  7.6% | 1  0.4% |
| (2) planning and structuring of tasks | 105  38.2% | 57  20.7% | 80  29.1% | 31  11.3% | 2  0.7% |
| (3) flexibility | 50  17.9% | 69  24.7% | 115  41.2% | 45  16.1% | 0  0.0% |
| (4) applying expertise | 129  47.6% | 71  26.2% | 50  18.5% | 21  7.7% | 0  0.0% |
| (5) competence to judge and decide | 110  39.9% | 68  24.6% | 69  25.0% | 29  10.5% | 0  0.0% |
| (6) endurance | 10  3.6% | 50  18.2% | 128  46.5% | 85  30.9% | 2  0.7% |
| (7) assertiveness | 63  22.9% | 67  24.4% | 105  38.2% | 40  14.5% | 0  0.0% |
| (8) contact with others | 90  32.7% | 88  32.0% | 84  30.5% | 13  4.7% | 0  0.0% |
| (9) group integration | 87  32.5% | 79  29.5% | 81  30.2% | 20  7.5% | 1  0.4% |
| (10) intimate relationships | 161  59.2% | 64  23.5% | 37  13.6% | 10  3.7% | 0  0.0% |
| (11) non-work activities | 114  41.9% | 101  37.1% | 44  16.2% | 11  4.0% | 2  0.7% |
| (12) self care | 136  49.3% | 73  26.4% | 53  19.2% | 14  5.1% | 0  0.0% |
| (13) mobility | 131  47.8% | 82  29.9% | 52  19.0% | 9  3.3% | 0  0.0% |
|  |  |  |  |  |  |
| Total ratings per category (n=3566) | 1302 | 959 | 948 | 349 | 8 |
| Distribution (%) | 37% | 27% | 27% | 10% | 0 |

**Table S5: Functional impairments related to alternative work (IFAP-2b)**

1. Ratings, mean, standard deviation

|  | **N** | **Mean** | **SD** |
| --- | --- | --- | --- |
| (1) adherence to regulations | 257 | 0.71 | 0.79 |
| (2) planning and structuring of tasks | 255 | 0.88 | 0.91 |
| (3) flexibility | 258 | 1.15 | 0.87 |
| (4) applying expertise | 254 | 0.60 | 0.73 |
| (5) competence to judge and decide | 258 | 0.78 | 0.87 |
| (6) endurance | 257 | 1.68 | 0.82 |
| (7) assertiveness | 256 | 1.11 | 0.91 |
| (8) contact with others | 260 | 0.83 | 0.81 |
| (9) group integration | 253 | 0.85 | 0.87 |
| (10) intimate relationships | 255 | 0.47 | 0.73 |
| (11) non-work activities | 253 | 0.71 | 0.82 |
| (12) self care | 254 | 0.72 | 0.89 |
| (13) mobility | 257 | 0.82 | 0.87 |
|  |  |  |  |
| IFAP-2b_global_ | 260 | 0.87 | 0.56 |

.

1. Reliability (intraclass correlation coefficient, ICC) and agreement (percentage of agreement and standard error of measurement, SEM) between four raters

| **IFAP-2b** | **ICC [95% CI]** | **Percentage of**  **Agreement (in %)** |
| --- | --- | --- |
| (1) regulations | 0.11 [0.01, 0.21] | 55.56 |
| (2) planning | 0.18 [0.07, 0.3] | 45.28 |
| (3) flexibility | 0.23 [0.11, 0.35] | 35.19 |
| (4) applying expertise | 0.13 [0.03, 0.24] | 68.0 |
| (5) competence to judge | 0.18 [0.07, 0.29] | 51.79 |
| (6) endurance | 0.2 [0.09, 0.32] | 26.42 |
| (7) assertiveness | 0.27 [0.15, 0.4] | 34.62 |
| (8) contact with others | 0.25 [0.14, 0.37] | 58.93 |
| (9) group integration | 0.24 [0.12, 0.37] | 48.98 |
| (10) intimate relationships | 0.21 [0.09, 0.34] | 39.58 |
| (11) non-work activities | 0.17 [0.06, 0.29] | 33.33 |
| (12) self-care | 0.17 [0.07, 0.27] | 89.29 |
| (13) mobility | 0.15 [0.05, 0.27] | 73.08 |
|  |  |  |
| Reliability and Agreement of IFAP-2b_global_ | **ICC [95% CI]** | **SEM [95% CI]** |
| **IFAP-2b_global_** | 0.26 [0.15, 0.38] | 0.49 [0.45, 0.52] |
| **WC_alt_ [%]** | 0.45 [0.31, 0.57] | 21.49 [19.06, 24.09] |

This table shows the reliability (ICC) and agreement (percentage of agreement between four raters) for the thirteen IFAP-2b domains and for the IFAP-2b_global_ score (expressed as SEM). Since we were interested in the IFAP categories that impacted on work capacity, we pooled the IFAP levels (0+1) and (3+4) to calculate percentage of agreement. The mean resp. median of the 13 IFAP-2a domains was 50.8% resp. 49.00% agreement. Both, ICCs and percentage of agreement of the individual domains, achieved only low values. For the global measures, IFAP-2b_global_  and WC_last_ reached poor to fair reliability values, while agreement (SEM) was poor in both parameters.

**Table S6: Distribution of ratings on functional impairment related to alternative work (IFAP-2b)**

|  | “0”  No  impairment | “1”  Mild  impairment | “2”  Moderate impairment | “3”  Severe impairment | “4”  Complete disability |
| --- | --- | --- | --- | --- | --- |
| (1) adherence to regulations | 122  47.5% | 94  36.6% | 35  13.6% | 6  2.3% | 0  0.0% |
| (2) planning and structuring of tasks | 108  42.4% | 81  31.8% | 55  21.6% | 10  3.9% | 1  0.4% |
| (3) flexibility | 64  24.8% | 107  41.5% | 71  27.5% | 16  6.2% | 0  0.0% |
| (4) applying expertise | 134  52.8% | 92  36.2% | 24  9.4% | 4  1.6% | 0  0.0% |
| (5) competence to judge and decide | 123  47.7% | 77  29.8% | 50  19.4% | 8  3.1% | 0  0.0% |
| (6) endurance | 17  6.6% | 88  34.2% | 113  44.0% | 38  14.8% | 1  0.4% |
| (7) assertiveness | 74  28.9% | 100  39.1% | 63  24.6% | 19  7.4% | 0  0.0% |
| (8) contact with others | 105  40.4% | 102  39.2% | 46  17.7% | 7  2.7% | 0  0.0% |
| (9) group integration | 104  41.1% | 94  37.2% | 43  17.0% | 12  4.7% | 0  0.0% |
| (10) intimate relationships | 164  64.3% | 66  25.9% | 20  7.8% | 5  2.0% | 0  0.0% |
| (11) non-work activities | 123  48.6% | 88  34.8% | 37  14.6% | 3  1.2% | 2  0.8% |
| (12) self care | 133  52.4% | 70  27.6% | 39  15.4% | 12  4.7% | 0  0.0% |
| (13) mobility | 116  45.1% | 80  31.1% | 52  20.2% | 9  3.5% | 0  0.0% |
|  |  |  |  |  |  |
| Total ratings per category (n=3325) | 1387 | 1139 | 648 | 149 | 2 |
| Distribution (%) | 42% | 34% | 19% | 4% | 0 |

**Supplement S7: Why training in functional assessment might have been insufficient**

This paragraph provides additional background information on why we cannot rule out the possibility that training in functional assessment may have been inadequate:

Empirical research in insurance medicine is scarce. For the RELY-program, we adopted a pragmatic approach, using available evidence and expert input rather than waiting for robust data. Based on a Dutch model, we planned five days of on-site training over four to six weeks, with participants assessing one case per week using the new method. However, during recruitment, psychiatrists were only willing to attend three on-site sessions of three hours each, supported by self-directed reading and, where possible, application to one or two real cases per month. We treated this reduced format of what is feasible as a real-world pilot. Given the time constraints, we proceeded within these limits, aiming to gain experience and refine the approach in future iterations.

**Supplement 8: The impact of expert certainty in their own judgment of residual work capacity**

**Question 1: Is expert variation in rWC judgments for the same claimant related to low certainty in their own judgment?** (Certainty measured on a scale from 0 = no certainty to 10 = high certainty)

Almost all experts expressed high certainty in their judgment of rWC: Certainty_rWC_last_ = 7.5 (mean; SD 1.8); Certainty_rWC_alt_ = 7.3 (mean; SD 1.8). Ratings below 5 were rare: Certainty_rWC_last_: 6.5%; Certainty_rWC_alt_: 5.9% of all ratings. This means, that despite low interrater agreement and reliability between experts, their certainty in their own judgment of rWC was high.

**Do experts who are more certain of their own rWC judgment achieve higher level of agreement on the four rWC judgments for the same claimant than experts who are less certain of their own judgment?**

A one-point increase in certainty reduced the deviation from the mean rWC_last_ per claimant by less than 1% rWC (mean 0.91% rWC, 95% CI -0.03; -1.80). This was a significant, but clinically negligible improvement in agreement. Improvement was even smaller with regards to rWC_alt_, where a 1-point increase in certainty reduced the deviation by 0.32% rWC (95% CI -1.11; 0.47).

**Conclusion:** More certainty does not mean better agreement

**Question 2: To what degree did certainty vary when experts acted as interviewer or observers?**

Comparing interviewing versus observing experts for their certainty in their own judgment of rWC showed that overall, experts were certain to very certain, whereby observers were slightly less certain than the interviewer (-0.85 points for rWC_last_ and - 0.77 points for rWC_alt_, respectively).

**Conclusion:** Minor differences between interviewers and observing raters in certainty of rWC judgments turned out to be negligible.

**Table Suppl 8**

|  | Interviewer | Observer | Difference in means  (95% CI) | p-value |
| --- | --- | --- | --- | --- |
| N observations | 69 | 191 |  |  |
| Certainty in rWC_last_ judgment  Mean (SD) | 8.17  (1.53) | 7.32  (1.88) | -0.85  (0.4; 1.31) | <0.001 |
|  |  |  |  |  |
| N observations | 62 | 178 |  |  |
| Certainty in rWC_alt_ judgment  Mean (SD) | 7.87  (1.88) | 7.1  (1.79) | -0.77  (0.23; 1.31) | 0.006 |
